# The baffle shape effects on natural convection flow and entropy generation in a nanofluid-filled permeable container with a magnetic field

**DOI:** 10.1038/s41598-024-53001-7

**Published:** 2024-01-31

**Authors:** Aissa Abderrahmane, Obai Younis, Abed Mourad, Houssem Laidoudi, Mowffaq Oreijah, Kamel Guedri, Sayed M. Tag

**Affiliations:** 1 Laboratoire de Physique Quantique de La Matière et Modélisation Mathématique (LPQ3M), University of Mascara, Mascara, Algeria; 2 Department of Mechanical Engineering, College of Engineering in Wadi Alddwasir, Prince Sattam Bin Abdulaziz University, Al-Kharj, Saudi Arabia; 3 Laboratory of Sciences and Marine Engineering, Faculty of Mechanical Engineering, USTO-MB, BP 1505, El-Menaouer, 31000 Oran, Algeria; 4 Mechanical Engineering Department, College of Engineering and Islamic Architecture, Umm Al-Qura University, P.O. Box 5555, 21955 Makkah, Saudi Arabia; 5 Future University in Egypt, New Cairo, 11835 Egypt

**Keywords:** Mechanical engineering, Applied mathematics

## Abstract

Enhancing heat transfer rates within enclosures is a topic of considerable interest since it has several technical applications. Most heat transfer research projects focus on increasing the heat transfer rates of thermal systems since this will raise the systems' total efficiency. The geometry of the enclosure might have a substantial impact on heat transfer rates. This research studies quantitatively the natural convection of a nanofluid in a complicated form geometry with many baffle configurations. The system's governing equations were addressed by the Galerkin Finite Element Method (GFEM). The main consideration was given to the effects of the following factors: The Darcy number (*Da*), which ranges from 10^–2^ to 10^–5^; the Hartmann number (*Ha*), which ranges from 0 to 100; the volumetric fraction (*ϕ*), which ranges from 0 to 0.08, and the Rayleigh number (*Ra*) (10^2^ to 10^6^). The results suggested that raising Ra increases heat transfer discharge, whereas raising *Ha* and *Da* decreases it. In terms of heat transmission, case 1 (the case with a wavenumber of 1 and the zigzag pointing outward) is determined to be the optimum cavity structure, as it obtained the highest mean Nusselt (Nu_avg_) number when compared to other cases. At the highest studied *Ra* number, growing (*ϕ*) from 0 to 0.8 improved *Nu*_avg_ by 25%, while growing *Da* from 10^–2^ to 10^–5^ and *Ha* from 0 to 100 declined *Nu*_avg_ by 57% and 48%, respectively. The reason for the improvement in the values of the (*Nu*) is due to the speed of fluid movement within the compartment. Also, the shape of fins plays a major role in strengthening and weakening thermal activity.

## Introduction

Understanding the mechanism of transmitting heat through different materials is essential to approve the performance of various engineering applications, especially mechanical, including energy storage units, solar systems, thermal management systems, and fuel cells. In the last two decades, it was suggested that the use of nanofluids (dissolved nanoparticles in a base fluid) and additional approaches could improve the heat transfer rate. Multiple research studies have shed light on the impact of many factors and techniques on the natural convection and heat transfer rate of nanoliquids in a variety of enclosures^1–4^. Buoyancy-driven flow is one of the key mechanisms in several thermal systems and devices. A variety of factors can influence it. Thus, to ameliorate the thermal processes of such devices, different strategies are of great necessity to be investigated, namely, the application of magnetic fields. In this respect, numerous investigations were reported in the literature to identify this subject^5–8^. Cao et al.^9^ examined the natural convection of nanoliquid within a square heat exchanger chamber supplied with two cylinders that functioned as heaters and coolers on both sides in the application of magnetic intensity. The obtained results declared that the Rayleigh number (*Ra*) number, magnetic intensity, and inclination angle have an effect on the nanoparticles' movement. Roy et al.^10^ performed a test on the natural heat convection of a hybrid nanoliquid enclosed in a bottom wall heated cage in addition to a subjected magnetic field. The findings demonstrated that the motion pattern was significantly altered when the magnetic field parameters were varied. Tayebi et al.^11^ undertook a numerical investigation of the Al_2_O_3_-H_2_O nanoliquid, which was surrounded by two circular cylinders, entropy generation, and thermal activity. The experiment was carried out with the application of magnetic intensity. This analysis revealed that factors such as *Ra*, fins' size, and *Ha* number have considerable effects on the thermal transfer within the annulus. Kargarsharifabad et al.^12^ explored the intervention of a magnetic field on the natural convection of nanoliquid (Cu-water) in a cubic chamber. This study's findings highlighted that adding nanoparticles of Cu to water ameliorates the ability to transfer heat when there isn't a magnetic field. However, Geridonmez et al.^13^ numerically assessed nanofluid flow and thermal process in a chamber with cross-fractional magnetic fields and a partial heater being applied. Their outcomes demonstrated that heat transmission was boosted with the *Ra*being boosted, the length of the partial heater, and the condensation of nanoparticles, whereas it was reduced when the force of Lorentz was increased. Molana et al.^14^ tested the properties of a steady inclined magnetic field on the thermal pattern of nanoliquid (Fe_3_O_4_/water) in a new shape of the porous cavity. Their findings suggested that the Hartmann number (*Ha*) augmentation limited heat transfer. Abdulkadhim et al.^15^ explored the heat transmission of Cu-H_2_Onanoliquid placed in a wavy-walled container equipped with a circular hot cylinder. Researchers have found that rising the *Ha* does not affect the Nusselt number *(Nu)*; however, it drastically reduced *Nu* at a relatively high *Ra* because of the restricted convection. A numerical investigation was done by Dogonshi et al.^16^ on magnetic nanofluid natural convection inside a permeable enclosure cooled from the outside and heated from the inside, with two additional walls being sequestered. According to the findings, the intensity of the convection might be affected by the Darcy number (*Da*) number, *Ra* number, *Ha* number, and inclination angle of magnetic intensity. Izadi et al.^17^ explored the influence of a diagonal magnetic field on the natural convection of a hybrid nanoliquid in a permeablechamber. Their outcomes showed that heat transmission was affected by the magnetic intensity in a non-monotonic manner. Sivaraj et al.^18^ digitally assessed the performance of a magnetic intensity on the convective flow of a ferrofluid in a chamber containing a vertical heated sheet. Outcomes showed that the rate of entropy generation witnessed a rise when an isothermal lamina was replaced by a non-uniformly warmed one, while it was reduced when an ideal inclined Lorentz force was set.

The natural convection process through porous materials has been adopted for heat transfer enhancement. Hence, countless papers were published on the convective motion of nanofluids within permeable medium^19–22^.Alsaberya et al.^23^ examined the thermal activity of an alumina-water nanoliquid soaked into a non-Darcy permeable medium, with nanoparticles being slithered in the base fluid. It was noticed that the nanoparticle concentration in the base water has a high degree of homogeneity. In addition, the Nu number increased as the *Da* number rose. Esfe et al.^24^ focused his research on 3D numerical simulations of nanofluid (CuO/water) heat transfer flow in a cubical chamber with firmly fixed porous fins attached to it. Results revealed that nanoparticle percentage affected the convective flow as well as the *Nu* number. Moreover, flow promptness dropped when porous fins were present. Kadhim et al.^25^ carried out an examination of free convection in a wavy wall container with a permeable material immersed in a hybrid nanofluid. Results revealed that the hybrid nanofluid was affected by the inclination angle. Furthermore, introducing nanoparticles boosted the convective flow between the walls. Cho et al.^26^ researched the thermal pattern of a nanofluid inside a permeable chamber with a semi-heated vertical side and wavy top and bottom walls. The obtained outcomes indicated that high rates of Da and Ra affected the convective flow indirectly. Mehryan et al.^27^ researched the heat transmission of nanofluids (Ag-MgO-water) inside a permeable room using the Local thermal non-equilibrium model. Their findings showed that the scattered hybrid nanoparticles' concentration of (Ag-MgO-water) reduced the natural convection. Khaled Al-Farhany et al.^28^ started research to dig into the heat transmission of a ferrofluid within a slanted heated porous container with two fins connected to the heated wall while the horizontal wall was sequestered. The end of the numerical assessment exposed that an augmentation in *Ra* and *Da* numbers and fins length led to a boost in the Nu number. Raizah et al.^29^ numerically scrutinized the convective nanofluid flow in a V-form chamber partially layered by a heterogeneous porous space. It was discovered from the results that the porous medium of the horizontal heterogeneous is the ideal state of porous space for a V-shaped hollow as the Nu number reached its culmination in it. Besides, the heat transmission is enhanced as the Ra number rises. Baghsaz et al.^30^ conducted a study on the convective flow of nanoliquid (Al_2_O_3_/water) in a porous space under the effect of nanoparticle deposition. The outcomes of this study showed that low porosities and long cavity lengths led to an extended sedimentation time. Furthermore, an augmentation in *Ra* and *Da* values resulted in a heat transmission boost. Izadi et al.^31^ launched an investigation on the free convection between two horizontal cylinders using the heat transport of water-diamond, water–silicon dioxide nanofluids, and water-copper within a porous space. It was noticed that water-diamond marked the greatest increase in heat transfer, while the water–silicon dioxide marked the total contrast. Abdulkadhim et al.^32^ simulated the thermal pattern of Ag nanofluid in a cold wavy container with two sides. The right one contained an Ag nanofluid, while the left one had an appeased porous space with the same nanofluid. According to the findings, the interior cylinder and the thickness of the porous layer influenced heat transmission.

Extended methods have been studied to enhance nanofluids' natural convection efficiency, such as incorporating baffles on the sides of enclosures^33,34^. Ma et al.^35^ tested the influence of magnetic intensity on the thermal behavior of a nanoliquid in a baffled U-shaped container. The findings showed that a lower Ha number led to a more noticeable magnetic field effect on heat transmission. Moreover, higher aspect ratios caused a substantial thermal transfer enhancement due to the *Ra* number influence. Naseri Nia et al.^36^ explored the natural convection of Cu-H_2_Onanoliquid inside a baffled L-shaped container, comparing the results to those of the L shape without a baffle. The outcomes indicated that adding a baffle improved heat transfer. Keramat et al.^37^ conducted a study on the buoyancy-driven flow of alumina/water nanofluid in a baffled H-shaped container with heated top and bottom walls and two less heated sidewalls. In the end, it was noticed that heat transport was enhanced due to the high *Ra* number and the temperature rise. Armaghani et al.^38^ numerically analyzed the thermal transfer of water-alumina nanoliquid within a baffled L-shaped chamber. The findings revealed two facts: (1) Raising the aspect ratio improves heat transfer. (2) A lengthy baffle enhances the natural convection indirectly. Nayak et al.^39^ tested the effect of Double-diffusive natural convection on the thermal patterns of a hybrid nanoliquid in a C-shaped chamber with two wavy baffles. It was noted that a rise in Rayleigh and Lewis numbers, as well as the wavy baffle's capacity, affected streamline density. Another result showed that the heat transport rate increased at any wavy baffles capacity. Al-Farhany et al.^40^ researched the impact of a slanted magnetic field on a nanoliquid-satiated porous medium in a baffled U-shaped container. The findings revealed that natural convection boosted with the *Ra* number, *Da* number, and nanoparticles rate boost, whereas the *Ha* number had the opposite impact.

According to the literature study, only a few researchers have used a baffle in nanofluid flow and looked into its consequences. Particularly, there is no indication that a zigzag baffle with multiple shapes was ever implemented. This study's main objective is to determine if adding a narrow baffle to divide fluid flow into parts may improve natural convection. The baffle significantly impacts the heat exchangers' flow pattern and thermal transfer characteristics. As a result, this article examined the impacts of baffle form on the flow structure and temperature generation in a chamber of a baffled U-shaped fully loaded with nanofluid (graphene/water) and a porous space under the effects of the following elements: a magnetic field, the *Ra* number, solid volume percentage (*ϕ*), *Ha* number, and various baffle cases are the primary variables due to their effects on heat transport. The outcomes are examined in terms of streamlines, entropy distribution, Bejan number (*Be*), average Nusselt number (*Nu*_*avg*_), and isotherms. The findings of this research can be applied in the thermal design of magnetic intensity elements and cooling systems for electronic equipment. We chose this type of shape because it is found in many engineering applications.

The new idea in this research is to clarify the performance of the shape of the zigzagged fins and their vertical position inside the chamber. Indeed, this effect is manifested in the quality of the thermal activity of the fluid as well as the behavior of the fluid. In addition to this, the results and analyses of this work can be exploited in developing thermal insulation systems, and they can also be used in compiling academic works related to heat transfer.

## The physical model

**Figure 1:**
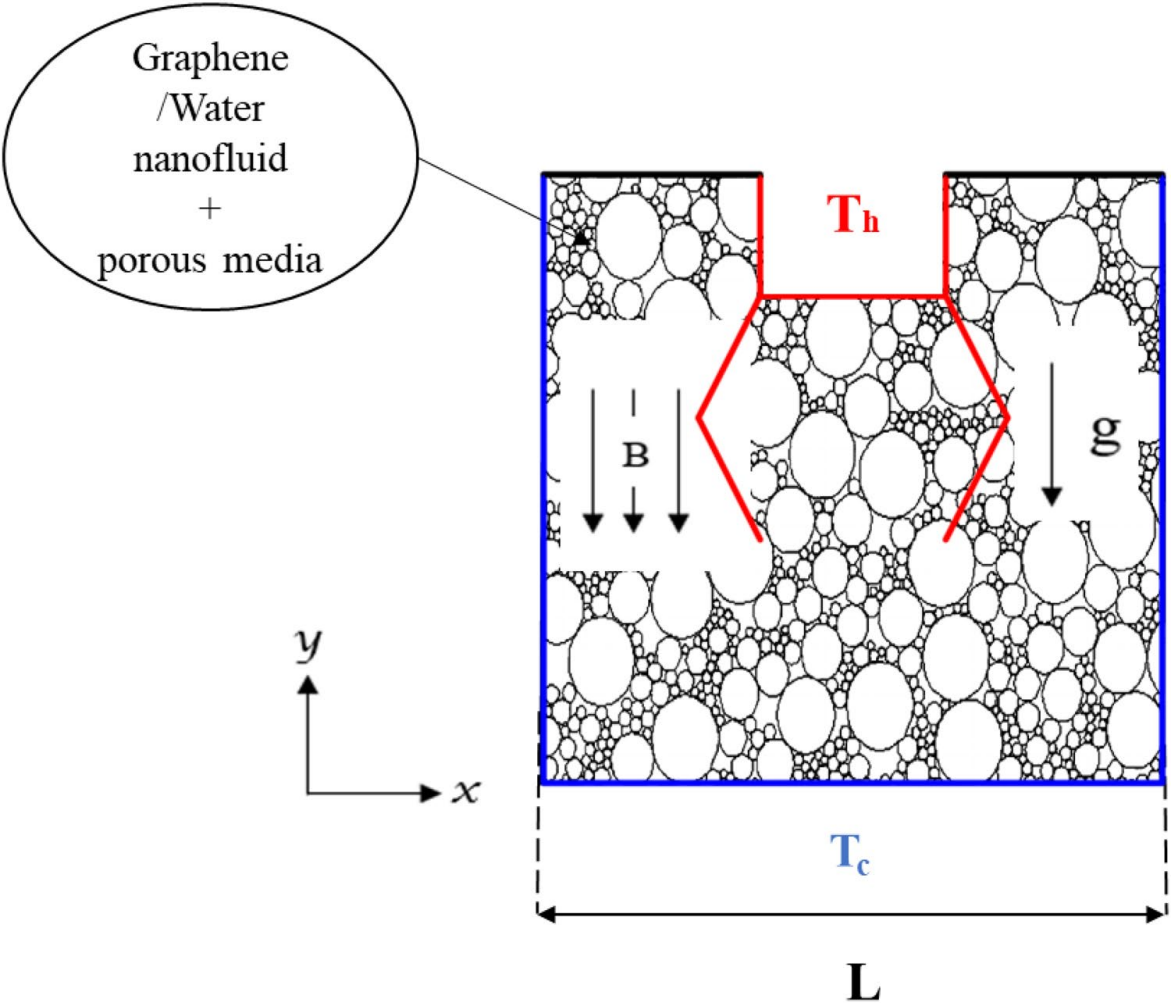
The physical domain.

The physical framework of the current study is depicted in Fig. 1. A Newtonian graphene nanofluid is loaded inside the permeable cavity with two internal corrugated hot baffles. The Forchhei-mer-Brinkman extended Darcy model is used to describe the permeable media around the cavity. The top cavity walls are adiabatic, side and bottom walls are preserved at a low temperature (*Tc*), while the corrugated baffles are heated (*Th*). To investigate the graphene nanoliquid and entropy in a U-shaped room by arranging two wavy baffles. The influence of Ra,*ϕ*, *Ha*, wave number on streamlines, isotherms, entropy distribution, mean Bejan number (*Be*), and Nusselt numbers (*Nu*) are well discussed. The generation of entropy as one of the vitally important elements is taken into account. Two baffles of symmetrical zigzag numbers are emplaced in the U-shaped chamber; the different scenarios are represented in Table 1. These hot zigzag baffles may have variable wave number (b), but their length (a) is assumed to be constant. These assumptions include 2D, incompressible, steady-state, and laminar motion, among others.

**Table 1:** The different investigated scenarios of hot zigzag baffles.

Case 1	Case 1.2
	
Case 2	Case 2.2
	
Case 3	Case 3.2
	

In this investigation, graphene and water were used as nanoparticles and base fluid, respectively; their respective thermophysical characteristics are reported in Table 2.

**Table 2:** Thermophysical characteristics of nanofluid(graphene/ water)^41^.

Properties	$\rho$(kg/m^3^)	*C*_*p*_ (J/kg K)	K (W/m K)	σ (S/m)	β (K^−1^)
Graphene	2250	2100	2500	10^7^	
Water	997.1	4179	0.613	5.5 × 10^−6^	21 × 10^−5^

## Mathematical formulations and boundary conditions

### Partial equations

The governing equations for mass, momentum, and energy of the situation under investigation may be defined using the following dimensional form based on the presumptions indicated above:

Continuity:

1 $$\frac{\partial \left(u\right)}{\partial x}+\frac{\partial \left(v\right)}{\partial y}=0$$

Momentum along x-direction:

2 $$\begin{array}{cc}\frac{1}{{\varepsilon }^{2}}\left(u\frac{\partial u}{\partial x}+v\frac{\partial u}{\partial y}\right)=& -\frac{1}{{\rho }_{\text{nf }}}\frac{\partial P}{\partial x}+\frac{{v}_{\text{nf }}}{\varepsilon }\left(\frac{{\partial }^{2}u}{\partial {x}^{2}}+\frac{{\partial }^{2}u}{\partial {y}^{2}}\right)-{\nu }_{{\text{nf}}}\frac{u}{K}-\frac{{F}_{{\text{c}}}}{\sqrt{K}}u\sqrt{{u}^{2}+{v}^{2}}+\frac{{\sigma }_{{\text{nf}}}}{{\rho }_{{\text{nf}}}}{B}_{0}^{2}\\ & \end{array}$$

Momentum along y-direction:

3 $$\begin{array}{cc}\left(u\frac{\partial v}{\partial x}+v\frac{\partial v}{\partial y}\right)=& -\frac{1}{{\rho }_{\text{nf}}}\frac{\partial P}{\partial y}+\frac{{v}_{\text{hf}}}{\varepsilon}\left(\frac{{\partial}^{2}v}{\partial {x}^{2}}+\frac{{\partial }^{2}v}{\partial {y}^{2}}\right)-{\nu }_{{\text{nf}}}\frac{v}{K}-\frac{{F}_{{\text{c}}}}{\sqrt{K}}v\sqrt{{u}^{2}+{v}^{2}}+{\beta}_{{\text{nf}}}g\left(T-{T}_{{\text{Avg}}}\right)+\frac{{\sigma}_{{\text{nf}}}}{{\rho }_{{\text{nf}}}}{B}_{0}^{2}.\end{array}$$

Energy*:*

4 $$\begin{array}{ll}& u\frac{\partial T}{\partial x}+v\frac{\partial T}{\partial y}={\alpha }_{{\text{m}}}\left(\frac{{\partial }^{2}T}{\partial {x}^{2}}+\frac{{\partial }^{2}T}{\partial {y}^{2}}\right)\\ & +\frac{{\varepsilon }_{{\text{p}}}{\mu }_{{\text{nf}}}}{{\left(\rho {c}_{{\text{p}}}\right)}_{{\text{nf}}}}\left\{\frac{1}{K}\left({u}^{2}+{v}^{2}\right)+\left(2\left[{\left(\frac{\partial u}{\partial x}\right)}^{2}+{\left(\frac{\partial v}{\partial y}\right)}^{2}\right]+{\left[\frac{\partial u}{\partial y}+\frac{\partial v}{\partial x}\right]}^{2}\right)\right\},\end{array}$$

where ${\alpha }_{{\text{m}}}=\frac{{k}_{{\text{m}}}}{{\left(\rho {c}_{{\text{p}}}\right)}_{{\text{nf}}}}$ is the thermal diffusiveness of the nanoliquid, ${k}_{{\text{m}}}=\left(1-{\varepsilon }_{{\text{p}}}\right){k}_{{\text{s}}}+{\varepsilon }_{{\text{p}}}{k}_{{\text{nf}}}$ is the thermal conductivity of the mixture effective parameter, and ${F}_{{\text{c}}}=\frac{1.75}{\sqrt{150{\varepsilon }_{{\text{p}}}^{3}}}$ denotes the Forchheimer coefficient, ${\varepsilon }_{{\text{p}}}$ is the porosity of the medium and ${d}_{{\text{m}}}$ is spherical-shaped particles.

$$K=\frac{{\varepsilon }_{{\text{p}}}^{3}{d}_{{\text{m}}}^{2}}{150{\left(1-{\varepsilon }_{{\text{n}}}\right)}^{2}}.$$

The dimensionless form of the basic equations was deduced by the following:

5 $$P=\frac{\left(p+{\rho }_{bf}{g}_{y}\right){L}^{2}}{{\rho }_{bf}{\alpha }_{bf}^{2}},V=\frac{vL}{{\alpha }_{bf}},U=\frac{uL}{{\alpha }_{bf}},X=\frac{\chi }{L},\theta =\frac{T-{T}_{f}}{{T}_{h}-{T}_{f}},Y=\frac{y}{L}.$$

Dimensionless numbers

6 $${\text{Da}}=\frac{\uplambda }{{{\text{L}}}^{2}}\left(\mathrm{Darcy number}\right)$$

7 $${\text{Pr}}=\frac{{{\text{v}}}_{{\text{fl}}}}{{\mathrm{\alpha }}_{{\text{fl}}}}(\mathrm{Prandtl number})$$

8 $$Ra=\frac{g{\beta }_{fl}({T}_{h}-{T}_{c}){L}^{3}}{{\alpha }_{fl}{v}_{fl}}\left(Rayleigh number\right)$$

9 $${\text{Ha}}=BL\sqrt{\frac{\sigma }{\mu }} (\mathrm{Hartmann number})$$

The following parameters characterize the graphene/water nanoliquid^41^:

10 $${\rho }_{nf}=(1-\varphi ){\rho }_{fl}+\varphi {\rho }_{s}$$

11 $${\left(\rho {c}_{p}\right)}_{nf}=(1-\phi ){\left(\rho {c}_{p}\right)}_{fl}+\phi {\left(\rho {c}_{p}\right)}_{s}$$

12 $${\left(\rho \beta \right)}_{nf}=(1-\varphi )(\rho \beta {)}_{fl}+\varphi (\rho \beta {)}_{s}$$

13 $$\frac{{k}_{nf}}{{k}_{fl}}=\frac{{k}_{s}+2{k}_{fl}-2\phi ({k}_{fl}-{k}_{s})}{{k}_{s}+2{k}_{fl}+2\phi ({k}_{fl}-{k}_{s})}$$

14 $${\mu }_{fl}=\frac{{\mu }_{fl}}{(1-\varphi {)}^{2.5}}$$

Finally, the partial equations become^8^:

15 $$\frac{\partial \left(U\right)}{\partial X}+\frac{\partial \left(V\right)}{\partial Y}=0$$

16 $$\begin{array}{cc}\frac{1}{{\varepsilon }^{2}}\frac{{\rho }_{\text{nf }}}{{\rho }_{\text{bf }}}\left(U\frac{\partial U}{\partial X}+V\frac{\partial U}{\partial Y}\right)=-\frac{{v}_{{\text{nf}}}}{{v}_{{\text{f}}}}\frac{{\text{Pr}}}{{\text{Da}}\sqrt{{\text{Ra}}}}U-\frac{{F}_{{\text{c}}}}{\sqrt{{\text{Da}}}}\sqrt{{u}^{2}+{v}^{2}}U+\frac{{\sigma }_{{\text{nf}}}}{{\rho }_{{\text{nf}}}}\frac{{\rho }_{{\text{f}}}}{{\rho }_{{\text{nf}}}}\frac{{{\text{PrHa}}}^{2}}{{\varepsilon }_{{\text{p}}}\sqrt{{\text{Ra}}}}{\text{U}},& \\ & \end{array}$$

17 $$\begin{array}{cc}\frac{1}{{\varepsilon }^{2}}\frac{{\rho }_{\text{nf }}}{{\rho }_{f}}\left(U\frac{\partial V}{\partial X}+V\frac{\partial V}{\partial Y}\right)=-\frac{{v}_{{\text{nf}}}}{{v}_{{\text{f}}}}\frac{{\text{Pr}}}{{\text{Da}}\sqrt{{\text{Ra}}}}V-\frac{{F}_{{\text{c}}}}{\sqrt{{\text{Da}}}}\sqrt{{u}^{2}+{v}^{2}}V+{\text{Pr}}\frac{{\beta }_{{\text{nf}}}}{{\beta }_{{\text{f}}}}g\theta +\frac{{\sigma }_{{\text{nf}}}}{{\rho }_{{\text{nf}}}}\frac{{\rho }_{{\text{f}}}}{{\rho }_{{\text{nf}}}}\frac{{{\text{Pra}}}^{2}}{{\varepsilon }_{{\text{p}}}\sqrt{{\text{Ra}}}}V,& \\ & \\ & \end{array}$$

18 $$U\frac{\partial \theta }{\partial X}+V\frac{\partial \theta }{\partial Y}=\frac{1}{\sqrt{{\text{Ra}}}}{\alpha }_{{\text{m}}}\frac{{\left(\rho {c}_{{\text{p}}}\right)}_{{\text{f}}}}{{k}_{{\text{f}}}}\left(\frac{{\partial }^{2}\theta }{\partial {X}^{2}}+\frac{{\partial }^{2}\theta }{\partial {Y}^{2}}\right)+\sqrt{{\text{Ra}}}\left(\frac{{\varepsilon }_{{\text{p}}}{\alpha }_{{\text{f}}}}{\left({T}_{{\text{h}}}-{T}_{{\text{c}}}\right)\cdot K}\right)\frac{{\mu }_{{\text{nf}}}}{{\left(\rho {c}_{{\text{p}}}\right)}_{{\text{nf}}}}\left\{\left({U}^{2}+{V}^{2}\right)+\frac{{\text{Da}}}{{\varepsilon }_{{\text{p}}}}\left(2\left[{\left(\frac{\partial U}{\partial X}\right)}^{2}+{\left(\frac{\partial V}{\partial Y}\right)}^{2}\right]+{\left[\frac{\partial U}{\partial Y}+\frac{\partial V}{\partial X}\right]}^{2}\right),\right.$$

#### Non-dimensional entropy generation

The size of the local entropy output is obtained by combining the merging flow and forces advanced. Non-dimensional local entropy output is set as follows in the process of heat convection when a magnetic field is present (Woods [49]).

19 $$\begin{array}{ll}{S}_{gen}=& \frac{{k}_{nf}}{{k}_{bf}}\left[{\left(\frac{\partial \theta }{\partial X}\right)}^{2}+{\left(\frac{\partial \theta }{\partial Y}\right)}^{2}\right]\\ & +\chi \frac{{\mu }_{\text{nf }}}{{\mu }_{bf}}\left\{\left({U}^{2}+{V}^{2}\right)+Da\left[2{\left(\frac{\partial U}{\partial X}\right)}^{2}+2{\left(\frac{\partial V}{\partial Y}\right)}^{2}+{\left(\frac{\partial U}{\partial Y}+\frac{\partial V}{\partial X}\right)}^{2}\right]\right\}\\ & +\frac{{\sigma }_{nf}}{{\sigma }_{f}}\chi H{a}^{2}{V}^{2}\end{array}$$

20 $$\chi =\frac{{\mu }_{\text{nf }}{T}_{avg}}{{k}_{bf}K}{\left(\frac{{\alpha }_{bf}}{L\left({T}_{H}-{T}_{C}\right)}\right)}^{2},{T}_{avg}=\frac{{T}_{H}+{T}_{C}}{2}$$

### Boundary conditions

The streamlines equation reads:

21 $$\frac{{\partial }^{2}\psi }{\partial {X}^{2}}+\frac{{\partial }^{2}\psi }{\partial {Y}^{2}}=\frac{\partial U}{\partial Y}-\frac{\partial V}{\partial X}$$

In this research, the boundary conditions are as follows:

Along the cold surfaces:

22 $$\theta =0,U=0,V=0$$

Along the hot surfaces:

23 $$\theta =1,U=0,V=0$$

For the adiabatic surfaces:

24 $$\frac{\partial \theta }{\partial Y}=0,U=0,V=0$$

Nu _loc_ and Nu _avg_ of the heated surfaces are respectively expressed as:

25 $$N{u}_{loc}={\left.-\frac{{k}_{eff}}{{k}_{fl}}\frac{\partial {\theta }_{po}}{\partial Y}\right\}}_{Y=0}$$

26 $$N{u}_{avg}={\int }_{0}^{1}N{u}_{loc}dX$$

Finally, GFEM was invented to solve the abovementioned fundamental Eqs. (15), (16), (17) and suitable boundary conditions (21), (22), (23). The Galerkin weighted residual approach was utilized to transform these fundamental equations into integral equations. The use of nanofluids improves the thermal characteristics of the normal fluid, and therefore, the secondary equations reflect this improvement. Mathematically, these equations do not cause a computational disturbance.

## Method validation and mesh independence

It is worth noting that this work was done through numerical simulations based mainly on solving differential equations. The methodology used is based on converting the differential equations modeled for fluid movement Eqs. (1), (2), (3) and heat transfer Eq. (4) into a matrix system. After this, the GFEM method intervenes to reach the solution by taking the initial boundary conditions (Eqs. (19), (20), (21), (22)) as a basis for the process. The solution process takes place in successive iterations, and the calculation stops when the solving error becomes less than 10^–8^. On the other hand, Eqs. (6), (7), (8), (9), (10), (11), (12), (13), (14) took into account the change in thermal properties of the fluid and the geometric medium studied.

Different grids were examined to arrive at a grid-independent conclusion, as demonstrated in Table 3. Since the disparities in Nu and ${\left|\psi \right|}_{max}$ produced by Grid No. 4 and Grid No. 3 are less than 0.01%; Mesh No. 3 is appropriate and eligible to generate grid-independent results and relatively lower computational time. Therefore, a grid size of 40,060 was used in this study.

**Table 3:** Nu_avg_ and ${\left|\psi \right|}_{max}$ for various grid elements.

Grid number	1	2	3	4	5
Grid resolutions	4226	6538	16,422	40,060	47,880
*Nu*_a vg_	2.4844	2.5929	2.8120	2.9443	2.9446
${\left|\psi \right|}_{max}$	9.7529	9.7648	9.7772	9.7846	9.7857

**Figure 2:**
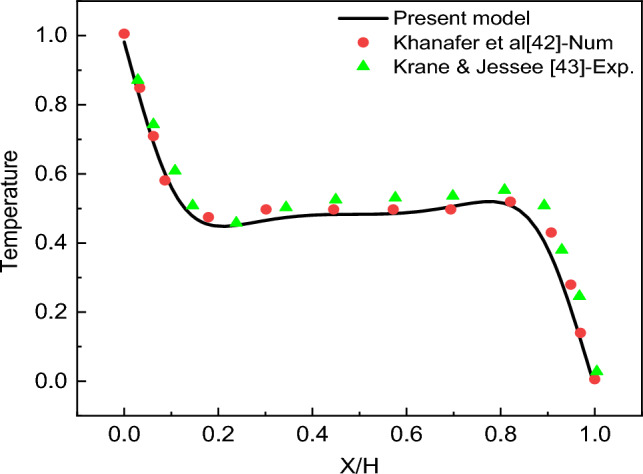
Validation of the present model against the results of Khanafer et al.^42^ and the experimental data of Krane and Jesse^43^.

The verification of the present code is performed by comparing numerically Khanfer^42^ and experimentally Krane and Jesee^43^. They investigated air flow in a cavity. The dimensionless temperature profile is plotted in Fig. 2, showing a very good agreement with a maximum deviation not exceeding 2%. Another comparison is performed for different Hartmann numbers, different volume percentages, and Ra = 10^5^. According to Table 4, the difference between the present work and that reported by Ghasemi et al.^44^ is acceptable.

**Table 4:** Validation of the present code for the MHD flow in a cavity^44^.

	Ha = 0		Ha = 30		Ha = 60	
	Ghasemi et al.^44^	Present work	Ghasemi et al.^44^	Present work	Ghasemi et al.^44^	Present work
Φ = 0	4.738	4.718	3.150	3.138	1.851	1.837
Φ = 0.02	4.820	4.803	3.138	3.117	1.831	1.814
Φ = 0.04	4.896	4.876	3.124	3.111	1.815	1.796
Φ = 0.06	4.968	4.939	3.108	3.189	1.806	1.791

## Results and discussion

The outcomes of this research aim to provide a comprehensive understanding of the movement of a nanofluid within a tightly closed space. The nanofluid inside the domain moves due to the thermal buoyancy force. That is, the liquid layers near the cold walls condense to become heavy, and this is what causes them to collapse to the bottom. On the other hand, nanofluid layers close to hot walls behave in the opposite direction, moving toward the top. Finally, we find the development of a circular flow within the studied space.

It is worth noting that the thermal patterns here are of the type of so-called buoyancy-driven flow. Therefore, the movement of nanofluid particles due to the thermal factor is controlled by the Rayleigh number. In this work, *Ra* ranges between 10^3^ to 10^6^. The *Da* number was chosen in the following range (10^−5^ to 10^−2^) to study the medium's permeability. *Ha* number was also considered in the range of 0 to 100 to know the influence of magnetic intensity on thermal transfer. These ranges were chosen since the simulations remain steady and laminar. In addition, some hot plankton of different shapes were installed on the heated part of the space to know their effect on the movement of the motion. The goal of including the influence of the magnetic force in our work is to find out if it is possible to control thermal activity through the intervention of this external force. The external force of the magnetic field is applied along the Y-axis. The solution of Maxwell's equation is obtained. The solution values are added to kinematic equations to reach the exact simulation.

**Figure 3:**
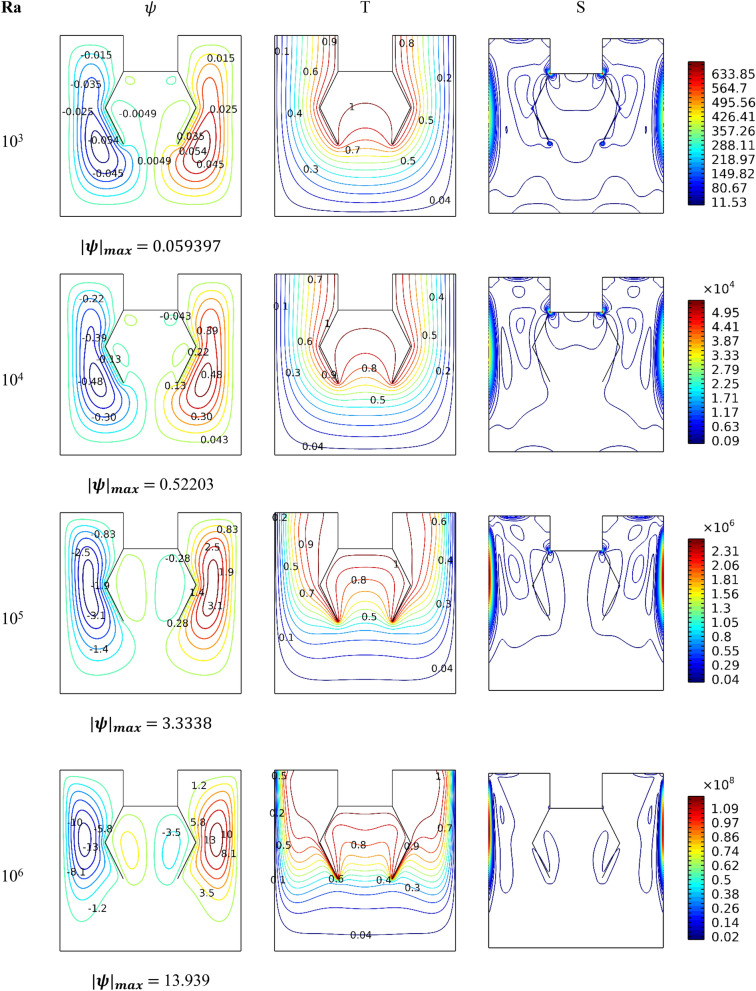
Influence of *Ra* number on dynamic and thermal patterns for *Da* = 10^–2^, *ϕ* = 0.04, and *Ha* = 0.

Figure 3 is inserted to clarify the impacts of Ra on the flow movement (streamlines), thermal pattern (dimensionless temperature), and the total entropy generation for *Da* = 10^–2^, *ϕ* = 0.04, and *Ha* = 0. As was discussed earlier, since the lateral walls are cold, the fluid layers close to them are heavier, and this causes them to move downwards. Whereas the hot fluid layers located near the hotlines become less dense and thus move upward. Therefore, through the streamlines, we notice the formation of two vortices within the space, the first on the right and moving in a clockwise direction, while the other on the left and moving counterclockwise. It is also noted that two small vortices are formed between the two lines. It is also illustrated that the velocity of the suspension movement grows with the increase in the value of *Ra*. Therefore, it is noted from the isotherms that the temperature gradient next to the heated surfaces augments with the increase in the number of *Ra*.

This indicates that the thermal transfer is strengthened in terms of the *Ra* number. The representative contours of entropy generation show that raising the value Ra increases its value. In addition, the maximum entropy generation is near the lateral walls because the flow velocity is considered in these zones. In general, raising the value of *Ra* strengthens the fluid movement, which results in stronger thermal activity.

**Figure 4:**
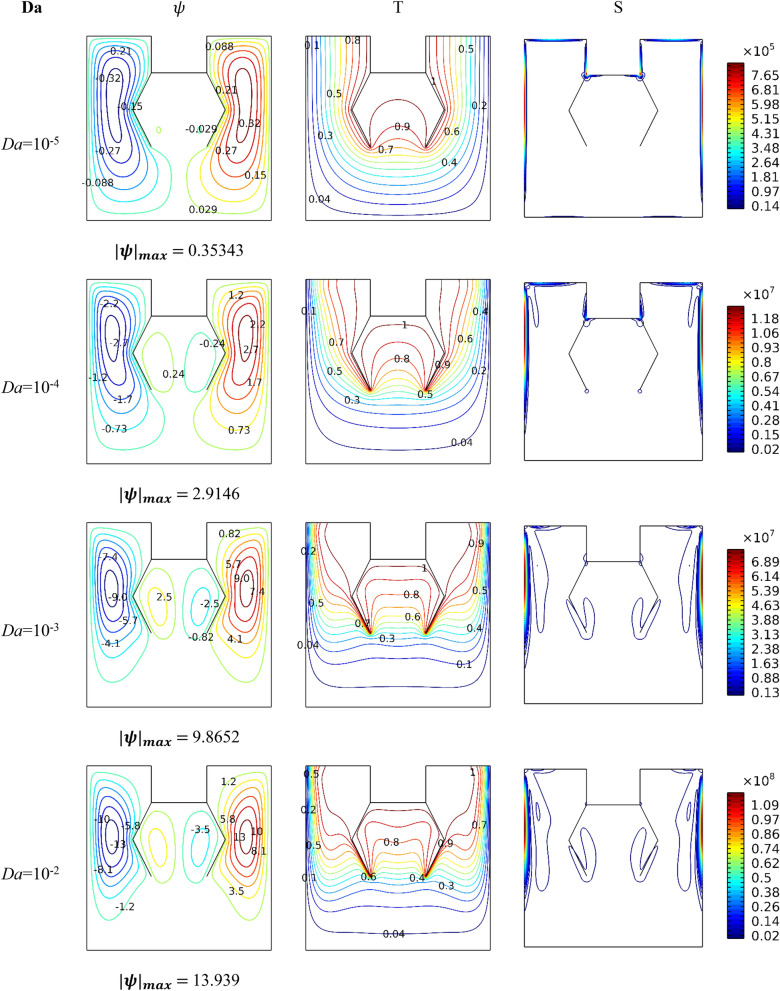
Impact of *Da* number on dynamic and thermal patterns for *Ra* = 10^6^,* ϕ* = 0.04, and *Ha* = 0.

Figure 4 shows the influence of the value of *Da* number on the movement of the nanofluid (pathlines), and heat dissipation (isotherms), as well as the entropy generation for *Ra* = 10^6^ and *Ha* = 0. The gradual increase in the value of the *Da* number means an augmentation in the medium's permeability, which makes the displacement of the suspension easier. Accordingly, we note that the velocity of the flow augments in terms of the *Da* number, i.e., the development of the vortices within the space also increases in terms of this number. In addition, we note that the temperature gradient next to the hot surfaces also grows in terms of the number *Da*, emphasizing the growth in heat transfer in terms of the growth in the value of the number *Da*. The same thing is observed regarding entropy generation, and whenever the medium allows the transfer of the nanofluid particles, this leads to an increase in entropy generation. Furthermore, the greatest value for entropy generation is always next to cold walls. In the end, it can be concluded that the greater the medium permeability, the easier and stronger the movement of the fluid.

**Figure 5:**
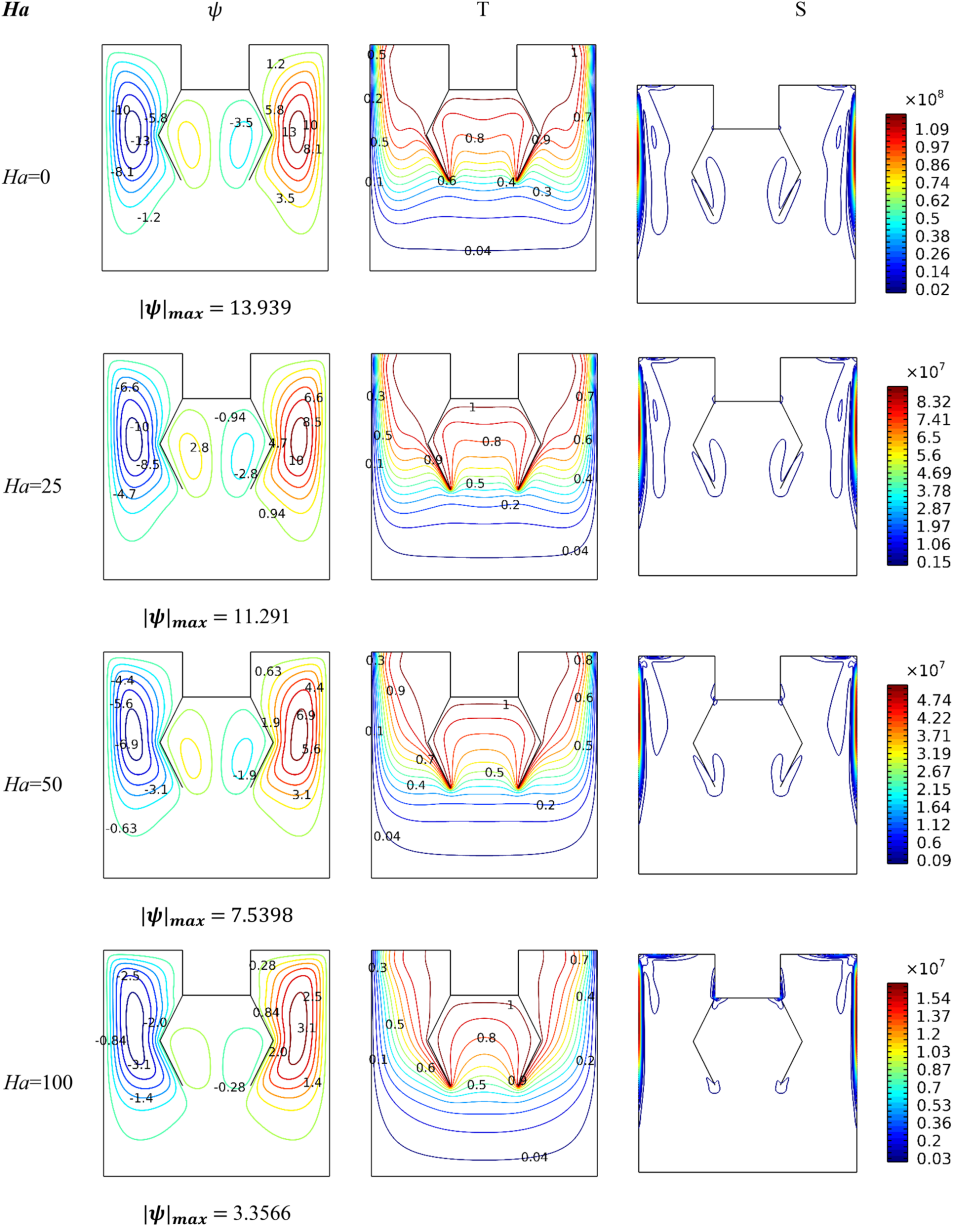
Impact of *Ha* number on dynamic and thermal patterns for* ϕ* = 0.04 *Da* = 10^−2^ and *Ra* = 10^6^.

Figure 5 presents the impact of the values of the number *Ha* on the thermal performance of the medium. Therefore, Fig. 5 shows isotherms, streamlines, and contours of entropy production for *Da* = 10^–2^, *ϕ* = 0.04, and *Ra* = 10^5^. We know that the presence of a magnetic intensity around moving ions creates a Lorentz force. In the current article, the impact of this force is opposite to the direction of the flow transmission, so the streamlines reveal a reduction in the value of flow speed in terms of the *Ha* number, which reflects its negative impact on the thermal distribution, i.e., the temperature gradient along the hot surfaces decreases in terms of the *Ha* number. Because the speed of the motion is decreasing in terms of the *Ha* number, the entropy generation contours are also declining.

**Figure 6:**
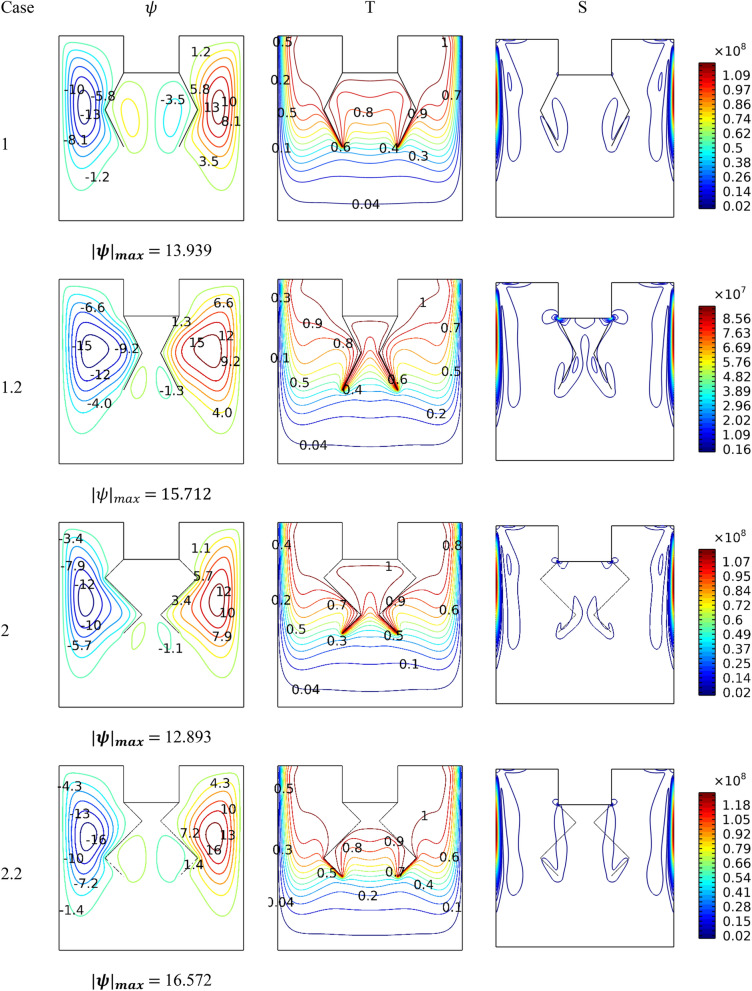

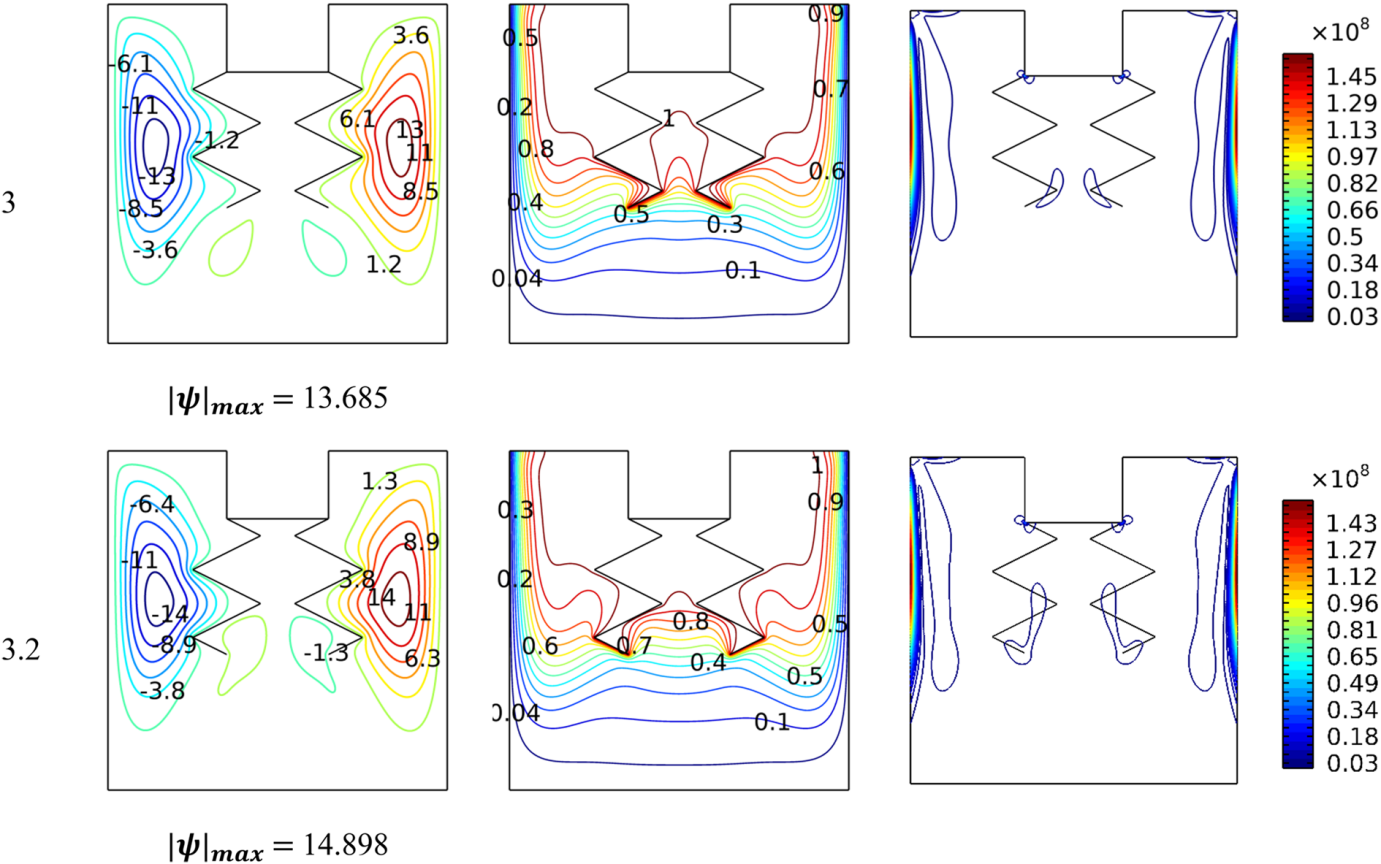
Influence of baffles' shape on dynamic and thermal patterns for *Ra* = 10^6^, *Da* = 0.01, and *Ha* = 0.

Figure 6 illustrates the performance of the shape of the hotlines for *Ha* = 0, *Ra* = 10^5^and*Da* = 0.01. It is observed that the shape of the lines affects the movement of the nanofluid and thus impacts the thermal activity of the entropy generation. In general, it is noted that the narrower the gap spacing between the two lines, the more this leads to a loss of ground in the velocity of the flow and, thus, a decrease in gradient temperature around the hot surfaces. Furthermore, the presence of hanging lines creates two small counter-rotating zones in the gap between the two lines. It is noticed that whenever the width of the gap decreases, this leads to the transfer of the two small vortices to the bottom.

**Figure 7:**
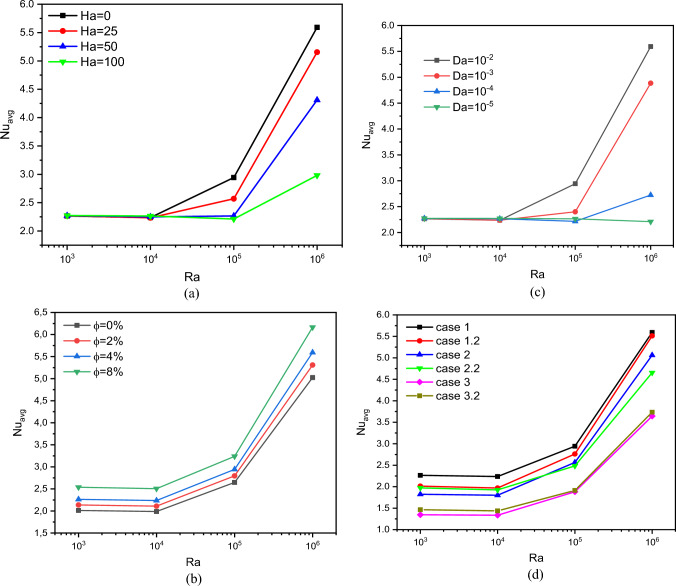
*Nu*_avg_ for different parameters.

Figure 7 summarizes the variations of the Nu_avg_ number with Ra for all cases and the whole range of *Da*, *Ha*, and *ϕ*. We recall that the Nu number means the ratio of the thermal transfer of the convective type of the fluid to the convective type of the fluid. Therefore, we can conclude that the higher value of *Nu* means that convective heat transfer increases. Figure 7a represents the impact of *Ha* and *Ra* numbers on *Nu* for case 1, *Da* = 10^–2^ and *ϕ* = 0.04. Contrary to the impact of Ra values, roughly seen that for all values of *Ra*, increasing the number of *Ha* negatively affected *Nu*. This decrease can be explained by the decline in the motion velocity due to the Lorentz force's resistance to the nanofluid's motion. Figure 7b is presented to help in understanding the impact of the nanoparticles concentration on *Nu* for case 1, *Ha* = 0 and *Da* = 10^–2^. The most important thing to notice here is that the higher the concentration of the nanoparticles, the higher the value of *Nu*. In this case, the reason for this augmentation is due to the enhancement in the thermal proprieties of the nanofluid. Figure 7c represents the results of the number *Nu* in terms of *Da* and *Ra* numbers for case 1, *Ha* = 0 and *ϕ* = 0.04. It is noticed that there is a clear impact of Da on Nu.

Briefly, the gradual increase in the number of *Da* means an expansion in the permeability, i.e., a reduction in the medium's resistance to the movement of nanofluid particles and, therefore, an increase in the heat transmission rate. Figure 7 (d) describes the impact of geometrical form on the values of *Nu* for *Da* = 0.01 and *Ha* = 0. It is seen that the value of *Nu* gradually decreases from case 1 to case 3. This decrease is mainly due to the following: we noted previously that the transition from case 1 to case 3 results in a decrease in the space between the two heated lines, and this makes the dynamic performance of the flow more difficult; accordingly, we notice a decrease in the heat transmission rate.

**Figure 8:**
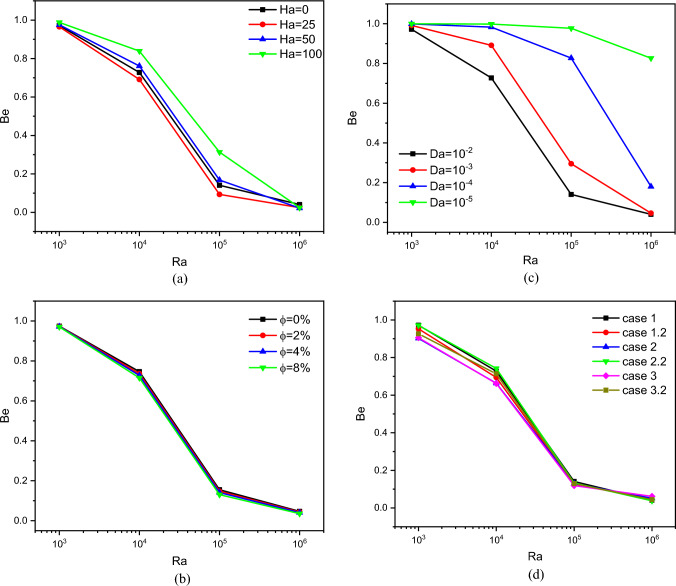
Bejan number for different parameters.

Figure 8 shows the development of the *Be* number with *Ra* for all cases and all ranges of *Da*, *Ha,* and *ϕ*. In the beginning, we mention that the number *Be* means the ratio of the resulting entropy generation due to thermal activity over the entropy generation caused by the movement of the fluid particles.

Figure 8a shows the evolution of the number Be in terms of *Ra* and *Ha* for case 1, Da = 10^–2^ and *ϕ* = 0.04. It is noted that the value of Be decreases in terms of Ra because of the movement of the flow growths with the thermal buoyancy factor (*Ra*). Conversely, the higher the number of *Ha*, the higher the value of Be due to the decline in the speed of the nanofluid particles. Figure 8b represents the distribution curves of the number Be in terms of *ϕ* and *Ra* for case 1, *Da* = 10^–2^ and *ϕ* = 0. We note that there is no effect of *ϕ* on Be because increasing nanoparticles' concentration does not affect the speed of the flow. Figure 8c depicts the relationship between the number *Da* and *Be* for case 1, *Ra* = 10^6^ and *ϕ* = 0. We notice that the higher the value of *Da*, the greater the value of Be. Furthermore, for *Da* = 10^–5^, all values of *Be* are limited between 1 and 0.8, indicating that the thermal source is predominant. Figure 8 (d) represents the impact of the geometry on the number *Be* for *Da* = 10^–2^, *ϕ* = 0, and *ϕ* = 0.04. We noticed that the impact of the geometry on *Be* is only for the small values of *Ra*.

## Conclusion

This work presented results of the free convection within a closed space with vertical zigzagged fins. This space also contains a nanofluid and foam with limited permeability. In addition to this, there is an external magnetic field penetrating the room. The research was conducted for these conditions: Da (10^–2^ to 10^–5^), *Ha* (0 to 100), *ϕ* (0 to 0.08), and Ra (10^2^ to 10^6^).

The study enabled us to reach these conclusions:

1. Raising the values of the following numbers (*Ra*, *Da,* and *ϕ*) increases the heat transfer of hot surfaces and flow velocity.
2. At the highest studied value of *Ra* number, increasing *ϕ* from 0 to 0.8 increased Nuavg by 25%, while increasing *Da* from 10^–2^ to 10^–5^ and *Ha* from 0 to 100 declined Nu_avg_ by 57% and 48%, respectively.
3. The magnetic field's presence resists the flow's movement and decreases the thermal activity rate.
4. The lengthening of the baffles impedes the movement of the fluid, which makes this method helpful in thermal insulation usage, and the type of geometric shape can be exploited in the technique for thermal insulation.
5. The smaller the space between the two lines, the lower the heat transfer of the hot surfaces.
6. The better the movement of the fluid within the space, the faster it transfers heat energy, and increasing the rate of added nanoparticles makes thermal activity stronger.

## Data Availability

All data generated or analyzed during this study are included in this published article.
